# Impact of tibial supplementary fixation in anterior cruciate ligament reconstruction for soft tissue auto and allografts: Modest enhancement in stability and increased incidence of pain: A systematic review and meta‐analysis

**DOI:** 10.1002/jeo2.70390

**Published:** 2025-08-05

**Authors:** Fuwen Zheng, Jiahao Gao, Chenyu Wang, Xu Zheng, Jinshuo Tang, Jinrui Zhang, Jianlin Zuo

**Affiliations:** ^1^ Department of Orthopedics China‐Japan Union Hospital of Jilin University Changchun Jilin China; ^2^ Wenzhou Medical College Affiliated Yueqing Hospital Yueqing Zhejiang China

**Keywords:** anterior cruciate ligament (ACL) reconstruction, hamstring, screw interference, supplementary fixation, tibial fixation

## Abstract

**Purpose:**

To evaluate whether the joint function, stability and safety of tibial supplementary fixation in anterior cruciate ligament reconstruction is superior compared with tibial screw fixation alone.

**Methods:**

PubMed, Cochrane Library, EMBASE and Web of Science were searched, tracking until 12 April 2025. Eligible studies included published randomized controlled trials (RCTs) and low‐risk cohort studies comparing clinical outcomes and complications between tibial screw interference with supplementary fixation (Group I) and tibial screw interference alone or with a sheath (Group II). RCTs were assessed using the Cochrane Risk of Bias tool, while cohort studies were evaluated with the Newcastle–Ottawa Scale and Methodological index for non‐randomized studies. Model selection (random or fixed‐effects) was based on data heterogeneity.

**Results:**

This meta‐analysis included eight studies with 943 patients (Group I: 386, Group II: 557). Group I showed no significant differences in side‐to‐side difference (SSD) in the sheath subgroup at 24 months, SSD <3 mm at 9.1 kg at 12 and 24 months, or manual maximum testing at 24 months, Pivot test at 8–12 and 24 months, Lachman test at 8–12 months, International Knee Documentation Committee objective and subjective score at 24 months compared to Group II. Group I demonstrated statistically significant reductions in SSD (mean difference: −1.02; 95% CI: −1.79 to −0.25; *p* = 0.009) in the no‐sheath subgroup and lower Lachman test positivity (odds ratio [OR] = 0.30; 95% confidence interval [CI]: 0.13–0.71; *p* = 0.01) at 24 months. Ligament retear rates were similar; however, Group I experienced a substantially higher incidence of kneeling pain (OR = 6.28; 95% CI: 1.86–2.25; *p* < 0.01), an outcome that could adversely affect patient comfort and long‐term functional recovery.

**Conclusion:**

Enhanced supplementary tibial fixation with soft tissue autografts and allografts offers similar joint function and a modest enhancement of stability compared to tibial interference screw fixation alone, but is associated with a higher incidence of pain.

**Level of Evidence:**

Level III, retrospective cohort studies have been analysed, alongside RCTs, and thus this is the level of evidence.

**Abbreviations**
ACL: anterior cruciate ligament reconstruction
BTB: bone–patellar tendon–bone
HT: hamstring tendon
IKDC: International Knee Documentation Committee
RCTs: randomized controlled trials
SSD: side‐to‐side difference

## INTRODUCTION

Anterior cruciate ligament (ACL) reconstruction is a widely performed surgical procedure designed to restore knee stability in patients with ACL injuries [36, 39]. Contrary to the distal femur, which has higher bone mineral density, proximal tibial‐sided fixation has been reported as the weakest point in ACL reconstruction [2, 31, 33]. Furthermore, the pullout force direction of the graft is mostly parallel to the tibial tunnel [7]. Therefore, enhancing the stiffness and strength of tibial‐side graft fixation is crucial to prevent early failure or loss of graft tension [34, 42].

Although clinical studies have investigated supplementary fixation modalities, such as interference screws, staples and spiked washer screws, to enhance tibial‐side fixation in ACL reconstruction, the results have been controversial [1, 12, 14, 20, 26, 27, 28, 42]. Earlier studies indicated that tibial supplementary fixation after ACL reconstruction could enhance graft stiffness and improve knee stability [20, 26, 28], and it was shown that hybrid fixation or Bone Bridge fixation had superior biomechanical performance compared with isolated extracortical fixation [31]. While recent studies have contested these findings [1, 12, 14, 27, 42]. Simultaneously, the use of some supplementary fixation devices may increase the risk of hardware prominence and pain, which could necessitate a second surgery for removal [5, 14, 22], resulting in additional surgical costs [21, 22].

Given the lack of consensus on the necessity of tibial supplementary fixation in ACL reconstruction, its use in treating ACL injuries remains a subject of debate. Thus, the purpose of this study was to evaluate the joint function, stability and safety of tibial supplementary fixation regimens compared to tibial screw fixation alone in ACL reconstruction for soft‐tissue grafts. The present study hypothesized that adding supplementary tibial fixation during ACL reconstruction with screw fixation on the tibial side would enhance anterior tibial stability but could increase the risk of local intolerance.

## METHODS

### Search strategy and eligibility criteria

This meta‐analysis followed the Preferred Reporting Items for Systematic Reviews and Meta‐Analyses (PRISMA) guidelines [30] and AMSTAR (Assessing the methodological quality of systematic reviews) Guidelines [37]. The protocol for the systematic review was registered with PROSPERO on 10 September 2023.

The PubMed, EMBASE, Cochrane Library and Web of Science databases were systematically searched using the keywords and combinations: (‘Anterior Cruciate Ligament’ AND ‘supplement*’ AND (‘second*’ OR ‘fixation*’)). These terms were utilized as both keywords (title or abstract words) and subject headings (e.g., MEDLINE medical subject headings) when appropriate. Data were retrieved on 24 March 2024, and updated on 12 April 2025 (Table S1). There is no start date restriction applied to capture the earliest relevant records. Additionally, the study screened the reference lists of the retrieved publications to identify additional studies. Searches were restricted to full‐text articles published in the English language.

The inclusion criteria were as follows: (1) study design: comparative, including randomized controlled trials (RCTs) and low‐risk non‐RCTs; (2) study subjects: patients who underwent primary ACL reconstruction surgery with soft‐tissue grafts; (3) outcome: the study compares the clinical outcomes and complications of treating ACL injuries at any stage using screw interference with supplementary tibial fixation (Group I) versus screw interference alone or with an added sheath (Group II) (A sheath, also called cannulated screw sleeve or graft protection device, is a cylindrical sleeve placed between the soft tissue graft and the interference screw during tibial fixation to avoid focal graft trauma and maintain uniform tunnel‐graft‐screw interface.) If possible, a subgroup analysis is performed within Group II to differentiate between screw interference alone and with the addition of a sheath. Abstracts, case reports, animal trials, reviews and unpublished studies were excluded.

### Study selection and data extraction

Two reviewers independently screened the titles and abstracts, followed by a full‐text assessment of the studies preliminarily included. Disagreements regarding the inclusion of the studies were resolved by consensus with the assistance of a third reviewer. Data were independently extracted by two reviewers using a predesigned data extraction form. This form, designed by the senior author, includes publication year, first author, study design, study inclusion and exclusion criteria, sample size, patient demographic data, follow‐up time, tendon types, graft numbers, tibial fixation methods and relevant scoring data of clinical outcomes and complications between Group I and Group II. Data were imported into Microsoft Excel (2019) and reviewed by the senior reviewer.

### Quality assessment and risk‐of‐bias assessment

Two independent researchers assessed the risk of bias in RCTs using the Cochrane Risk of Bias tool [19]. Each study was assessed using seven criteria that broadly covered the areas of random sequence generation, allocation concealment, blinding of participants and personnel, blinding of outcome assessment, incomplete outcome data, selective reporting, and other bias. The risk of bias within each domain was categorized as low, high or unclear.

Observational studies were assessed for risk of bias using the Newcastle–Ottawa Scale [40]. Each study was assessed across three categories: selection, comparability and outcome. A maximum of 9 points could be awarded, and a score of 6 points was the minimum threshold for the study to be considered of high quality [35]. Additionally, two investigators reviewed and graded each of the three included observational studies for quality by applying the Methodological Index for Non‐Randomized Studies (MINORS) scoring system. Each of the 12 MINORS items is scored 0 to 2, score 0 if not reported, 1 when reported with inadequacy, 2 when reported and adequate. A perfect score was 16 for non‐comparative studies and 24 for comparative studies. A score ≥ 16 points in comparative studies or a score ≥ 11 points in non‐comparative studies is considered high‐quality research [15, 38, 44].

Quality assessments of the studies were conducted independently by two reviewers, and any discrepancies were resolved through consensus. The certainty and strength of the evidence were assessed using the Grading of Recommendations, Assessment, Development and Evaluation (GRADE) criteria [4], which classify evidence from high to very low certainty.

### Statistical analysis

Statistical analyses of the data and risk assessments were conducted using STATA (version 18.0; Stata Corp LLC) software and Review Manager version 5.4.1 (The Cochrane Collaboration). The mean difference (MD) served as the effect index for continuous variables, using the inverse variance method, while the odds ratio (OR) was used as the effect index for dichotomous data, with the Mantel–Haenszel test applied. Additionally, 95% confidence intervals (CIs) were calculated for all variables, and heterogeneity was evaluated using the Higgins *I* ^2^ statistic. Two‐sided *p* < 0.05 was considered statistically significant. If the heterogeneity was low (*p* > 0.10 and *I* ^2^ < 50%), a fixed‐effects model was used for data analysis. Otherwise, a random effects model was used [17].

## RESULTS

### Search results

**Figure 1:**
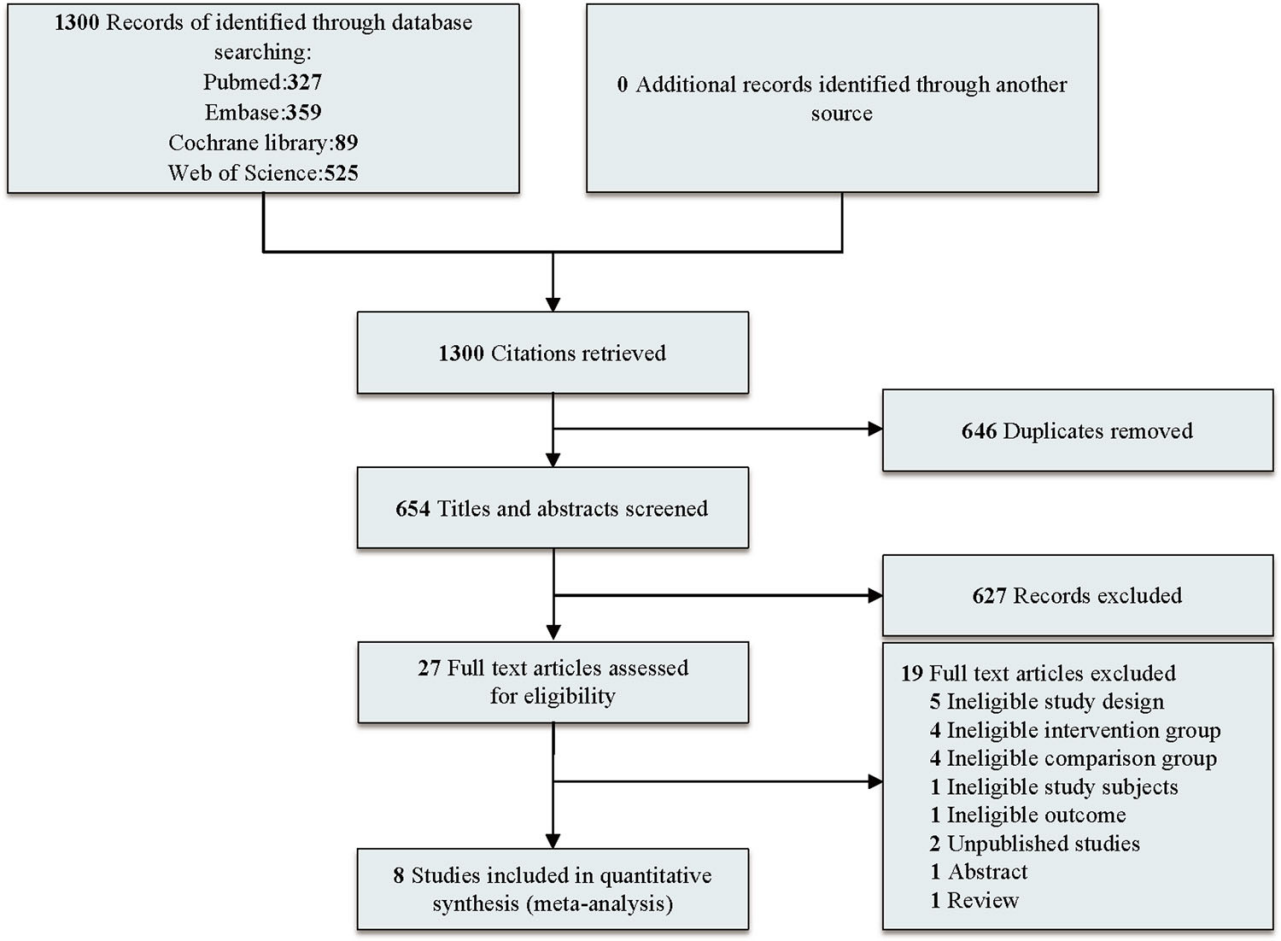
Flowchart of studies included in the analysis (last search: April 2025).

The review initially identified 1300 relevant studies through the searches of four medical databases, of which 27 were deemed potentially eligible after title and abstract reviews; of these, 19 were excluded for inappropriate study design for the aims of this review (5 studies), ineligible intervention (4 studies) or comparison groups (4 studies), incorrect subjects (1 study) or outcomes (1 study), unpublished status (2 studies), abstract‐only availability (1 study), and being a review article (1 study). A total of eight studies (five RCTs, three cohort studies) were finally included in the analysis [1, 12, 14, 20, 26, 27, 28, 42]. The PRISMA flowchart of the article‐selection process is presented in Figure 1.

### Study characteristics

The characteristics of all eligible trials are outlined in Table 1. The eight studies included a total of 943 ACL reconstruction patients (Group I: 386, Group II: 557), and the duration of follow‐up was extended from 8 to 24 months after ACL reconstruction. In all studies, tibial fixation methods were interference screws (absorbable or non‐absorbable) with/without supplementary staples, with two studies [12, 14] adding a sheath in groups without tibial supplementary fixation. Notably, each of the included studies detailed postoperative rehabilitation protocols following ACL reconstruction, with the primary aim of restoring a complete range of motion and muscle strength.

**Table 1:** Characteristics of the patients in the included studies [1, 12, 14, 20, 26, 27, 28, 42].

Author, year	Study design	The level of evidence	Tendon types, graft numbers, femoral and tibial fixation methods	Total patients	Age (years)		Gender (F/M)		Clinical outcomes	Follow‐up (months)
					Group I	Group II	Group I	Group II		
Hill et al., 2005	RCT	Ⅱ	The HT autograft (gracilis and semitendinosus tendons), 4 strands of graft, the left femoral graft fixation using a standard 7 × 25 mm RCI (Smith & Nephew Endoscopy Inc.) interference screw and the right using a reverse‐thread 7 × 25 mm RCI interference screw. **Group I**: 7 × 25 mm RCI interference screw fixation with a supplementary staple; **Group II:** 7 × 25 mm RCI interference screw tibial fixation.	48	32.5 (28–38)	25.8 (15–41)	21:0	27:0	①②③⑤⑥	24
Lim et al., 2009	Co, R	Ⅲ	The HT autograft (gracilis and semitendinosus tendons), 4 strands of graft, femoral graft fixation using an EndoButton CL (Smith & Nephew Inc.). **Group I**: BioRCI‐HA interference screw fixation (Smith & Nephew Inc.) and a supplementary staple (Smith & Nephew Richards); **Group II**: solitary BioRCI‐HA interference screw tibial fixation.	31	25.3 (17–41)	22.5 (17– 38)	0:15	0:16	①⑥	24
De Wall et al., 2011	RCT	Ⅰ	The HT autograft, 4 strands of graft, femoral graft fixation using a continuous loop EndoButton 15–35 mm in length (Smith & Nephew Endoscopy). **Group I**: 25 mm interference screw (diameter, 7‐9 mm) (RCI, Smith & Nephew) and 3 or 4 staples, which were inserted with a 13 × 20 mm Shapiro Stapilizer (3 M); **Group II**: polyethylene screw (diameter, 7–10 mm) and sheath (INTRAFIX, Depuy Mitek)	90	33 ± 10.3	34 ± 8.3	‐	‐	①④⑥	24
Noh et al., 2012	RCT	Ⅱ	Tendon Achilles allograft, 2 strands of graft, femoral graft fixation using an EndoButton CL (Smith & Nephew). **Group I**: 10 × 28 mm Bio‐Interference screw (Arthrex, Naples, FL) and a 4.5 mm bicortical screw with a washer; **Group II:** 10 × 28 mm Bio‐Interference screw.	71	22 (19–45)	23 (19–45)	‐	‐	①②③⑤⑥	24
Teo et al., 2017	Co, R	Ⅲ	The HT autograft (gracilis and semitendinosus tendons), 4 strands of graft, femoral graft fixation using continuous loop EndoButton with 15 mm loop (Smith & Nephew Endoscopy). **Group I**: 25 mm bioabsorbable polyethylene interference screw with a 7.94 × 23.22 mm regular fixation staple (Smith & Nephew Richards); **Group II:** 25 mm bioabsorbable polyethylene interference screw.	64	25.6 ± 8.2	24.2 ± 8.6	6:27	6:25	①②③⑥	12
Carulli et al., 2017	RCT	Ⅱ	The HT autograft, 4 strands of graft, femoral graft fixation using an EndoButton CL with a variable length of 15–35 mm (Smith & Nephew). **Group I:** 30 mm resorbable PLA interference screw (Smith & Nephew) with two 8 × 25 mm titanium (Ti) staples (Citieffe); **Group II:** 30 mm resorbable polylactic acid/tricalcium phosphate (PLA/TCP) screw (diameter, 6–10 mm) and sheath (diameter, 7–9 mm).	90	31.8 (18–44)	31.0 (16–42)	12:33	9:36	①④⑥	24
Mousavi et al., 2020	RCT	Ⅱ	The HT autograft, **NR**, **NR,** **Group I**: interference screw with a supplementary staple; **Group II:** interference screw.	52	31.81 ± 6.37	29.83 ± 6.33	15:9	20:8	②③⑥	8
Abudaqqa et al., 2023	Co, R	Ⅲ	The HT autograft (gracilis and semitendinosus tendons), 2–7 strands of graft, femoral graft fixation using a suspensory fixation with an Endobutton (Smith & Nephew Endoscopy). **Group I**: 25 mm absorbable interference screw with an 8‐mm metal staple; **Group II:** 25 mm absorbable interference screw.	497	30.61 ± 6.99	30.30 ± 7.24	1:166	2:328	⑥	12

*Note*: ① Side‐to‐side difference; ② Pivot test; ③ Lachman test; ④ IKDC Subjective Score; ⑤ IKDC Objective Grade; ⑥ Complications.

Abbreviations: Co, Cohort study; HT, hamstring tendon; IKDC, International Knee Documentation Committee; NR, not reported; R, retrospective study; RCT, randomised controlled trial.

### Risk‐of‐bias assessment

All five of the RCTs included were at low risk of bias, and none of the cohort studies scored less than 7 points in NOS or 16 points in MINORS (Tables S2–S4). Funnel plots could not assess publication bias because none of the meta‐analyses included more than 10 studies.

### Meta‐analyses

#### Ligament laxity evaluation

**Figure 2:**
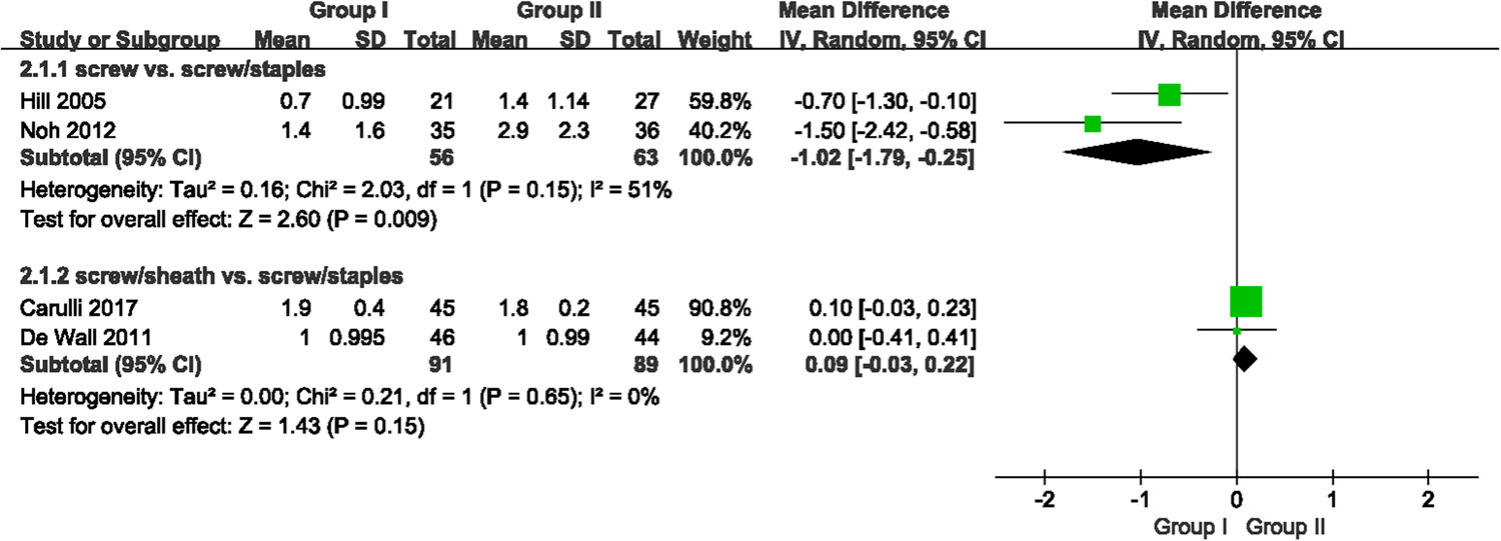
Forest plot of the subgroup results of sheath and no‐sheath at 24 months between Group I and Group II after ACL reconstruction (Group I: screw interference with supplementary fixation; Group II: screw interference alone). ACL, anterior cruciate ligament; CI, confidence interval; IV, inverse variance; SD, standard deviation.

Six studies measured the side‐to‐side difference (SSD) post‐ACL reconstruction, employing different devices: the KT‐2000 arthrometer in three studies [12, 26, 42], the KT‐1000 in two [12, 20], and the Telos stress device in another [28]. At the point of 24 months, the pooled results from two studies in the no‐sheath subgroup revealed a significantly lower SSD in Group I compared to that in Group II (MD: −1.02; 95% CI: −1.79 to −0.25; *p* = 0.009). Nevertheless, no significant SSD was observed between the two groups in the sheath subgroup (MD: 0.09; 95% CI: −0.03 to −0.22; *p* = 0.15) (Figure 2). Similarly, for the subgroup of patients with movement of <3 mm, no significant difference was found between two groups on testing at 9.1 kg (20 lb) at both 12 (OR: 1.07; 95% CI: 0.14–8.09; *p* = 0.95) and 24 months (OR: 2.95; 95% CI: 0.75–11.51; *p* = 0.12) (Figure S1), and on manual maximum testing at 24 months (OR: 2.83; 95% CI: 0.95–8.41; *p* = 0.06) (Figure S2).

### Clinical examinations

**Figure 3:**
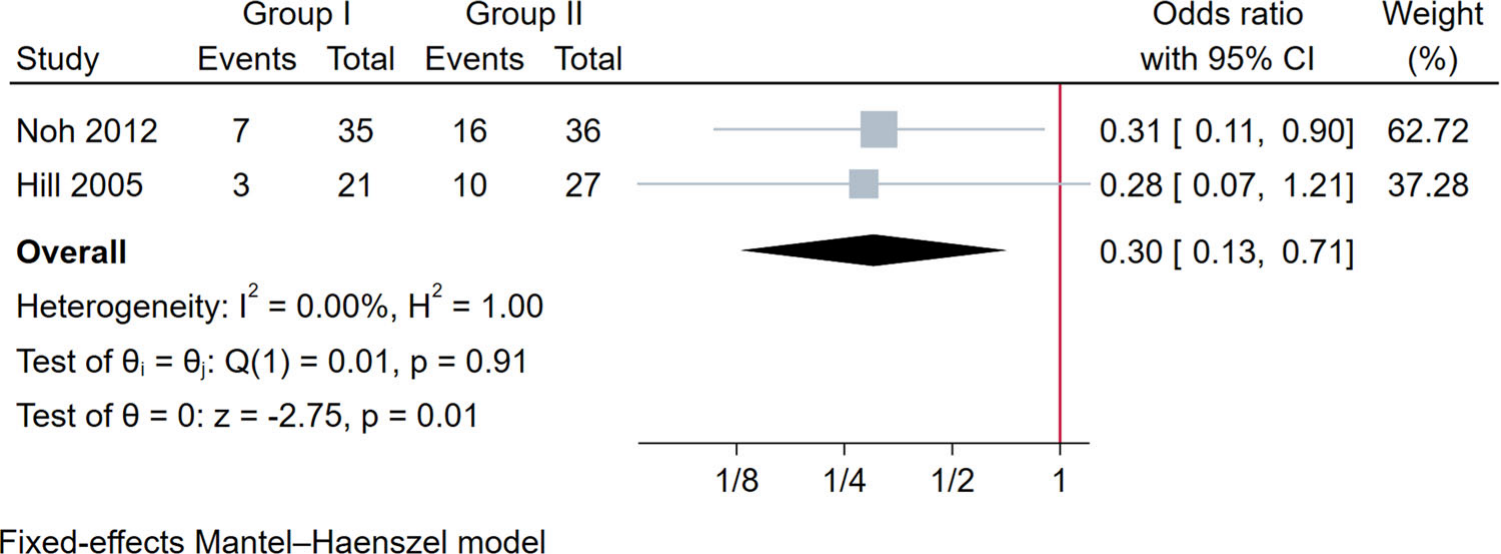
Forest plot of the results of the Lachman test at 24 months between Group I and Group II after ACL reconstruction (Group I: screw interference with supplementary fixation; Group II: screw interference alone). ACL, anterior cruciate ligament; CI, confidence interval.

A total of four studies recorded Pivot test outcomes at 8–12 and 24 months [20, 27, 28, 42]. The pooled results suggested no significant differences in outcomes between study groups following stratification by follow‐up at 8–12 months (OR: 1.40; 95% CI: 0.40–4.95; *p* = 0.60) and 24 months (OR: 0.35; 95% CI: 0.12–0.99; *p* = 0.05) (Figure S3). The Lachman test was assessed at 8–12 and 24 months in four studies following ACL reconstruction [20, 27, 28, 42]. For studies with 8‐ to 12‐month follow‐ups, no significant differences were observed in the proportion of patients with an abnormal grade (positive, 1+, or 2+) between the groups (OR: 0.82; 95% CI: 0.19–3.57; *p* = 0.79) (Figure S4). However, pooled data from two studies with 24‐month follow‐up showed that Group I had a significantly lower proportion of patients with an abnormal grade compared to Group II (OR = 0.30; 95% CI: 0.13–0.71; *p* = 0.01) (Figure 3).

### Functional outcomes

Postoperative knee function was evaluated using the International Knee Documentation Committee (IKDC) objective grade and subjective score in four studies with a follow‐up duration of 24 months [12, 14, 20, 28]. Pooled data revealed no significant difference in the proportion of patients with an abnormal grade (C or D) between the two groups on IKDC objective grade (OR: 0.47; 95% CI: 0.16–1.45; *p* = 0.19) (Figure S5), and on IKDC subjective score (MD: 1.42; 95% CI: −2.08 to 4.92; *p* = 0.43) (Figure S6).

### Major complications

**Figure 4:**
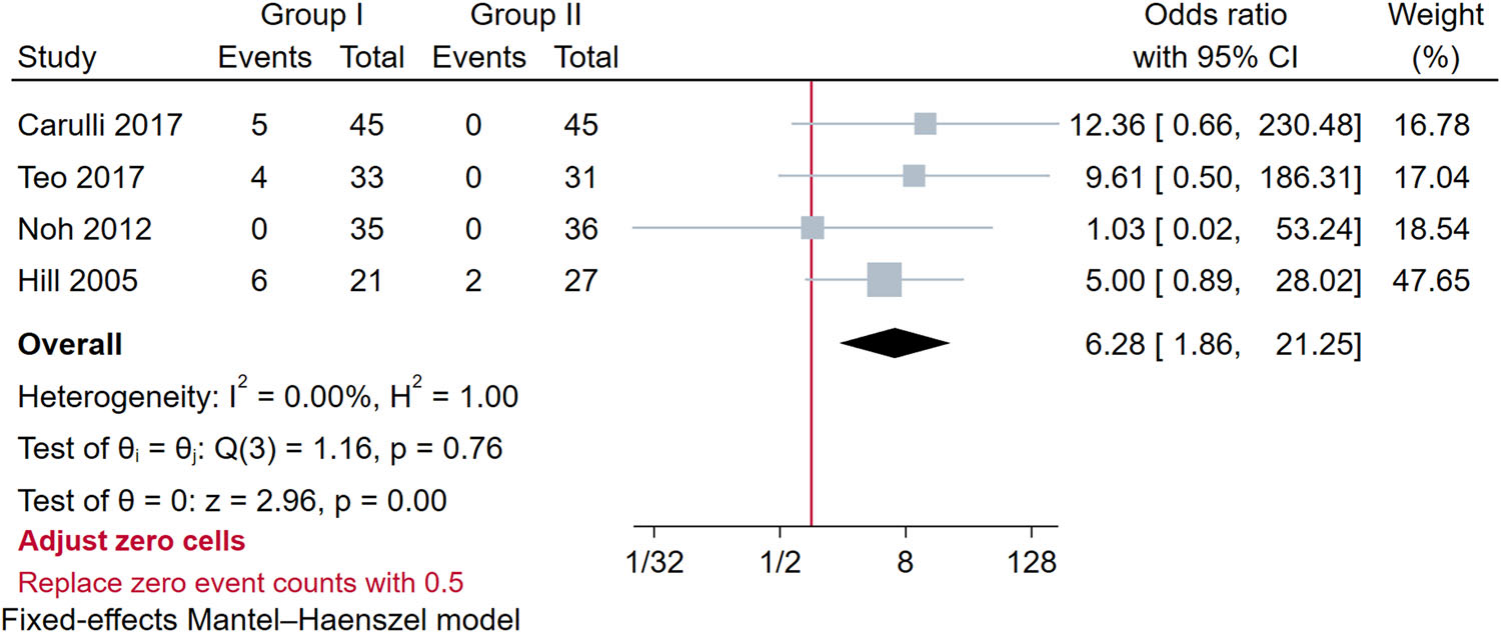
Forest plot of the results of kneeling pain between Group I and Group II after ACL reconstruction (Group I: screw interference with supplementary fixation; Group II: screw interference alone). ACL, anterior cruciate ligament; CI, confidence interval.

Adverse reactions were documented in all included studies [1, 12, 14, 20, 26, 27, 28, 42], with the most common complications being knee pain and ACL retear (Table S5). The pooled analysis [12, 20, 28, 42] showed that the incidence of kneeling pain was 11.2% in Group I and 1.4% in Group II after ACL reconstruction with a significant difference (OR = 6.28; 95% CI: 1.86–2.25; *p* = 0.00) (Figure 4). The pooled analysis [1, 12, 14, 27] revealed no significant difference in the probabilities of ACL retear between the sheath and no‐sheath subgroup (OR: 0.57; 95% CI: 0.24–1.35; *p* = 0.76) (Figure S7).

### Sensitivity analyses

Our sensitivity analyses confirmed the robustness of primary outcomes by utilizing random‐effects models versus fixed‐effect models, with effect magnitude stability, consistent directionality of effects. These findings support the methodological stability of our conclusions.

### Quality assessment

To facilitate GRADE certainty assessment, the OR was converted into a risk ratio (RR) by statistical analysis software (Table S6). Of the pooled data, the GRADE certainty of evidence was high for Pivot test, Lachman test at 24 months, kneeling pain and ACL retear (screw/sheath vs. screw/staples); moderate for SSD, the SSD subgroup (<3 mm) tested at 9.1 kg, manual maximum with 24‐month follow‐ups, and IKDC objective grade with 24‐month follow‐up; low and very low for five other measures.

## DISCUSSION

As the first systematic review and meta‐analysis specifically evaluating supplementary tibial fixation in ACL reconstruction using exclusively soft tissue autografts and allografts, this review found no significant differences between supplementary staple fixation and solitary tibial fixation with an interference screw in subgroup analyses of SSD with <3 mm movement, Pivot test, IKDC classification, and incidence of common complications, regardless of follow‐up duration. However, compared to Group II, Group I showed significantly improved SSD and Lachman test outcomes at a follow‐up duration of 24 months. These results may indicate that supplementary tibial fixation in ACL reconstruction may provide a modest enhancement in anterior tibial stability compared to using interference screws alone. Finally, findings showed that the differences in incidence of kneeling pain between the two groups were significant, while the differences in other adverse events between the two groups were minimal. These findings provide the first evidence‐based framework for surgical decision‐making in ACL reconstruction.

### Graft types and configurations

The majority of the included studies used hamstring tendon (HT) autografts, with the exception of one study that utilized an Achilles tendon allograft [28]. Ahn et al. [3] found that both grafts produced comparable clinical outcomes after a minimum 2‐year follow‐up in patients undergoing arthroscopic posterior cruciate ligament reconstruction. Among graft configurations, five studies designed four‐strand grafts from tendons [12, 14, 20, 26, 42], one used two‐strand grafts [28], another employed grafts ranging from two to seven strands [1], and one did not specify the graft configuration [27]. Evidence suggested that no significant differences in retear rates or functional recovery between four‐strand and other configurations [10]. And most included studies use four‐strand grafts; this is unlikely to be a significant concern. While double‐bundle techniques may offer theoretical advantages in rotational stability [23, 41], current evidence within the 2‐year follow‐up period showed no superiority over single‐bundle approaches [18].

### Femoral fixation strategies

Aperture techniques, such as using screws, and suspensory fixation, such as using Endobutton, were the primary femoral fixation methods [43, 46]. Six studies employed Endobuttons [1, 12, 14, 26, 28, 42], while only one used interference screws [20]. Another study did not provide details on femoral graft fixation [27]. Systematic reviews and meta‐analyses have consistently demonstrated equivalent outcomes in knee stability and clinical performance between these fixation modalities [11, 13, 16].

### Tibial fixation variables

Tibial interference screw diameters varied across studies based on the graft size of the HT autografts, though evidence suggests graft diameter does not directly correlate with postoperative stability [9].

Previously, biomechanical studies by Yıldırım et al. [47], Bauer et al. [6] and Lee et al. [25] on animal tibias found no significant differences between groups using interference screws with supplementary staples versus interference screws alone. In contrast, Laxdal et al. [24] reported benefits from adding supplementary staple fixation to the interference screw. Mousavi et al. [27] and Teo et al. [42] found no difference in laxity between Group I and Group II during short‐term (ranging from 8 to 12 months) follow‐ups in the included study. Surprisingly, at 24 months, the SSD and Lachman test results revealed significant differences between the two groups, suggesting that adding tibial supplementary fixation may improve long‐term knee stability. However, it should be noted that this conclusion is based on two studies [20, 28], with the *I* ^2^ of 51% heterogeneity in the no‐sheath subgroup and differences in the types of tendons used. The observed difference of 1 mm in SSD may not be clinically significant.

### Long‐term stability versus functional outcomes

While supplemental tibial fixation demonstrated improved long‐term knee stability at 24 months, functional outcomes (IKDC scores) showed no significant differences between groups at any follow‐up interval [27, 42]. This discordance suggests that enhanced biomechanical stability from supplementary fixation may not translate to measurable functional improvements in most patients.

### Complications and clinical implications

Although Group I demonstrated significantly better SSD and Lachman test results at the 24‐month follow‐up, supplemental tibial fixation was associated with a higher incidence of kneeling pain and symptomatic hardware requiring removal. So, when performing tibial supplementary fixation, its potential drawbacks, such as increased pain at the tibial fixation site, which may incur additional time and cost for staple removal, should be carefully considered.

Anterior knee pain has multiple contributing factors, including nerve injury or histological changes at the graft harvest site. Different graft harvest techniques, such as the two‐incision patellar tendon graft harvest technique, were shown to reduce kneeling pain [8]. And it is shown that some different tendons autograft demonstrated different outcomes regarding anterior knee pain [32]. Furthermore, different rehabilitation protocols also contributed to postoperative knee pain [29]. Therefore, although this meta‐analysis demonstrated an increased incidence of postoperative kneeling pain with tibial supplementary fixation, the heterogeneity among the included studies regarding graft types, rehabilitation protocols and harvest techniques necessitates further investigation to definitively establish its relationship with kneeling pain.

And no differences were observed in ACL retear rates and other complications between fixation strategies. Hence, it is believed that adding supplementary tibial fixation may be appropriate for specific populations, such as women with reduced bone density, as suggested by Hill et al. [20]. However, this improvement is at the cost of increased kneeling pain [20].

### Strengths and future directions

This analysis incorporated rigorous methodology, including GRADE evidence assessment, yielding moderate‐to‐high quality conclusions across major outcomes. However, limitations persist due to heterogeneity in graft configurations, fixation techniques and limited sufficient RCTs and high‐quality level III evidence.

### Limitations

Nevertheless, several limitations of this meta‐analysis should be noted. First, while there are eight included studies, the pooled data make many comparisons, but typically only pool two studies due to the heterogeneity in the outcomes reported in the individual studies, which may restrict the robustness of the findings and impact the effectiveness threshold [45]. Second, the inclusion of cohort studies may lead to unmeasured and residual confounding, which is reflected in the low certainty of evidence as evaluated by GRADE for non‐RCTs. Third, the present study did not assess the impact of graft selection on clinical outcomes of ACL reconstruction, as only one study used an Achilles tendon allograft, making subgroup analysis by graft type difficult [28]. Fourth, some of the studies included exclusively participants of the same gender, which may affect the generalizability and robustness of the outcomes [20, 26]. Finally, given that the maximum follow‐up in this analysis was 24 months, future investigations should prioritize long‐term outcomes (>5–10 years) to comprehensively evaluate delayed complications, graft failure rates and hardware‐related symptoms. To optimize clinical decision‐making, subsequent research would be better to: (1) standardize graft preparation protocols across institutions; (2) conduct formal cost‐effectiveness analyses of supplementary fixation techniques; and (3) establish prospective registries tracking functional outcomes beyond 5 years.

## CONCLUSION

Overall, the findings of this systematic review and meta‐analysis indicated that incorporating supplementary tibial fixation in ACL reconstruction for soft tissue auto and allografts with interference screws may provide an enhancement in anterior tibial stability compared to using interference screws alone, though these differences are modest and their clinical significance remains uncertain. However, the potential for an increased incidence of pain should also not be ignored.

## AUTHOR CONTRIBUTIONS

*Concept and design*: Jianlin Zuo. *Acquisition, analysis or interpretation of data*: Fuwen Zheng, Jiahao Gao and Jinrui Zhang. *Drafting of the manuscript*: Fuwen Zheng and Jiahao Gao. *Critical revision of the manuscript for important intellectual content*: All authors. *Statistical analysis*: Jiahao Gao, Fuwen Zheng and Jinrui Zhang. *Obtained funding*: Jinrui Zhang. *Administrative, technical or material support*: Jianlin Zuo. *Supervision*: Jinrui Zhang and Jianlin Zuo.

## CONFLICT OF INTEREST STATEMENT

The authors declare no conflicts of interest.

## ETHICS STATEMENT

The ethics statement is not available.

## Supporting information

Supporting information.

## Data Availability

The data that support the findings of this study are available from the corresponding author upon reasonable request.

## References

[1] Abudaqqa RY , Abed AR , Toubasi AA , Hantouly AT , Al Mas AJ , Abushaaban FA , et al. Stand‐alone tibial interference screw fixation and tibial interference screw plus tibial staple fixation produce comparable outcomes after primary anterior cruciate ligament reconstruction using hamstring autografts. Arthrosc Sports Med Rehabil. 2023;5:100810. 37881192 10.1016/j.asmr.2023.100810 PMC10594557

[2] Aga C , Rasmussen MT , Smith SD , Jansson KS , LaPrade RF , Engebretsen L , et al. Biomechanical comparison of interference screws and combination screw and sheath devices for soft tissue anterior cruciazte ligament reconstruction on the tibial side. Am J Sports Med. 2013;41:841–848. 23404085 10.1177/0363546512474968

[3] Ahn JH , Yoo JC , Wang JH . Posterior cruciate ligament reconstruction: double‐loop hamstring tendon autograft versus Achilles tendon allograft—clinical results of a minimum 2‐year follow‐up. Arthroscopy. 2005;21:965–969. 16084294 10.1016/j.arthro.2005.05.004

[4] Atkins D , Best D , Briss PA , Eccles M , Falck‐Ytter Y , Flottorp S , et al. Grading quality of evidence and strength of recommendations. BMJ. 2004;328:1490. 15205295 10.1136/bmj.328.7454.1490 PMC428525

[5] Bachmaier S , Smith PA , Argintar EH , Chahla J , Higgins LD , Wijdicks CA . Independent suture augmentation with all‐inside anterior cruciate ligament reconstruction reduces peak loads on soft‐tissue graft. A biomechanical full‐construct study. Arthroscopy. 2022;38:88–98. 34655766 10.1016/j.arthro.2021.09.032

[6] Bauer LAR , Alberti HAA , Corotti VGP , Franco APGO , Stieven Filho E , Cunha LAM . Análise biomecânica da dupla fixação de enxerto tendinoso em tíbia porcina—uso de parafuso de interferência e agrafe. Rev Bras Ortop. 2018;53:564–569. 30245995 10.1016/j.rboe.2018.07.003 PMC6147759

[7] Beattie KA , Boulos P , Duryea J , O'Neill J , Pui M , Gordon CL , et al. The relationships between bone mineral density in the spine, hip, distal femur and proximal tibia and medial minimum joint space width in the knees of healthy females. Osteoarthritis Cartilage. 2005;13:872–878. 16154772 10.1016/j.joca.2005.06.010

[8] Beaufils P , Gaudot F , Drain O , Boisrenoult P , Pujol N . Mini‐invasive technique for bone patellar tendon bone harvesting: its superiority in reducing anterior knee pain following ACL reconstruction. Curr Rev Musculoskelet Med. 2011;4:45–51. 21594690 10.1007/s12178-011-9077-8 PMC3097322

[9] Bedi A , Maak T , Musahl V , O'Loughlin P , Choi D , Citak M , et al. Effect of tunnel position and graft size in single‐bundle anterior cruciate ligament reconstruction: an evaluation of time‐zero knee stability. Arthroscopy. 2011;27:1543–1551. 21705174 10.1016/j.arthro.2011.03.079

[10] Bouché PA , Lefevre N , Bohu Y , Gerometta A , Meyer A , Grimaud O , et al. Comparison of the retear rate 2 years after ACL reconstruction with the Tape Locking Screw short graft system and the STG technique: a case control study with propensity score matching. Orthop Traumatol Surg Res. 2024;110:103848. 38408559 10.1016/j.otsr.2024.103848

[11] Browning 3rd WM , MA Kluczynski , Curatolo C , Marzo JM . Suspensory versus aperture fixation of a quadrupled hamstring tendon autograft in anterior cruciate ligament reconstruction: a meta‐analysis. Am J Sports Med. 2017;45:2418–2427. 28068159 10.1177/0363546516680995

[12] Carulli C , Matassi F , Soderi S , Sirleo L , Munz G , Innocenti M . Resorbable screw and sheath versus resorbable interference screw and staples for ACL reconstruction: a comparison of two tibial fixation methods. Knee Surg Sports Traumatol Arthrosc. 2017;25:1264–1271. 27120190 10.1007/s00167-016-4135-9

[13] Colvin A , Sharma C , Parides M , Glashow J . What is the best femoral fixation of hamstring autografts in anterior cruciate ligament reconstruction?: a meta‐analysis. Clin Orthop Relat Res. 2011;469:1075–1081. 21063817 10.1007/s11999-010-1662-4 PMC3048246

[14] De Wall M , Scholes CJ , Patel S , Coolican MRJ , Parker DA . Tibial fixation in anterior cruciate ligament reconstruction: a prospective randomized study comparing metal interference screw and staples with a centrally placed polyethylene screw and sheath. Am J Sports Med. 2011;39:1858–1864. 21622815 10.1177/0363546511406234

[15] Elkhadem A , Mickan S , Richards D . Adverse events of surgical extrusion in treatment for crown‐root and cervical root fractures: a systematic review of case series/reports. Dent Traumatol. 2014;30:1–14. 23796195 10.1111/edt.12051

[16] Elmholt SB , Nielsen TG , Lind M . Fixed‐loop vs. adjustable‐loop cortical button devices for femoral fixation in ACL reconstruction—a systematic review and meta‐analysis. J Exp Orthop. 2022;9:106. 36269424 10.1186/s40634-022-00544-1 PMC9587170

[17] Gao J , Ma Y , Tang J , Zhang J , Zuo J . Efficacy and safety of platelet‐rich plasma and hyaluronic acid combination therapy for knee osteoarthritis: a systematic review and meta‐analysis. Arch Orthop Trauma Surg. 2024;144:3947–3967. 38972025 10.1007/s00402-024-05442-y

[18] Gobbi A , Whyte GP . Anatomic double‐bundle and single‐bundle ACL reconstruction after ACL rupture did not differ for quality of life at 2 years. J Bone Jt Surg. 2019;101:943. 10.2106/JBJS.19.00179 31094990

[19] Higgins JPT , Altman DG , Gotzsche PC , Juni P , Moher D , Oxman AD , et al. The Cochrane Collaboration's tool for assessing risk of bias in randomised trials. BMJ. 2011;343:d5928. 22008217 10.1136/bmj.d5928 PMC3196245

[20] Hill PF , Russell VJ , Salmon LJ , Pinczewski LA . The influence of supplementary tibial fixation on laxity measurements after anterior cruciate ligament reconstruction with hamstring tendons in female patients. Am J Sports Med. 2005;33:94–101. 15611004 10.1177/0363546504268036

[21] Hurwit DJ , Habet NA , Meade JD , Berk AN , Young BL , Odum S , et al. Biomechanical comparison of Tibial‐sided supplemental fixation techniques in Bone‐Patellar Tendon‐Bone anterior cruciate ligament reconstruction. Knee. 2023;41:66–71. 36638705 10.1016/j.knee.2022.12.005

[22] Joshi A , Basukala B , Singh N , Rijal N , Gurung S , Nepal S , et al. Implantless supplementary fixation of anterior cruciate ligament in tibia with “make and use” anchor. Arthrosc Tech. 2024;13:102825. 38435267 10.1016/j.eats.2023.09.001 PMC10907883

[23] Koga H , Muneta T , Yagishita K , Watanabe T , Mochizuki T , Horie M , et al. Mid‐ to long‐term results of single‐bundle versus double‐bundle anterior cruciate ligament reconstruction: randomized controlled trial. Arthroscopy. 2015;31:69–76. 25242512 10.1016/j.arthro.2014.07.020

[24] Laxdal G , Kartus J , Eriksson BI , Faxen E , Sernert N , Karlsson J . Biodegradable and metallic interference screws in anterior cruciate ligament reconstruction surgery using hamstring tendon grafts: prospective randomized study of radiographic results and clinical outcome. Am J Sports Med. 2006;34:1574–1580. 16685087 10.1177/0363546506288014

[25] Lee JJ , Otarodifard K , Jun BJ , McGarry MH , Hatch 3rd, GF , Lee TQ . Is supplementary fixation necessary in anterior cruciate ligament reconstructions? Am J Sports Med. 2011;39:360–365. 21220546 10.1177/0363546510390434

[26] Lim CT , Tan KJ , Chuan AK . Clinical stability and outcome of supplementing tibial fixation with a staple for ACL reconstruction using hamstring tendons. Current Orthopaedic Practice. 2009;20:660–664.

[27] Mousavi S , Masoumi O , Akbariaghdam H , Mohammadsharifi G . Investigation of hamstring tendon graft fixation for the reconstruction of anterior cruciate ligament using interference screw merely or in combination with supplementary staple: a clinical trial. Adv Biomed Res. 2020;9:52. 33457335 10.4103/abr.abr_257_19 PMC7792865

[28] Noh JH , Yang BG , Yi SR , Roh YH , Lee JS . Hybrid tibial fixation for anterior cruciate ligament reconstruction with Achilles tendon allograft. Arthroscopy. 2012;28:1540–1546. 22732367 10.1016/j.arthro.2012.03.012

[29] Ordahan B , Küçükşen S , Tuncay I , Sallı A , Uǧurlu H . The effect of proprioception exercises on functional status in patients with anterior cruciate ligament reconstruction. J Back Musculoskeletal Rehabil. 2015;28:531–537. 10.3233/BMR-140553 26406302

[30] Page MJ , McKenzie JE , Bossuyt PM , Boutron I , Hoffmann TC , Mulrow CD , et al. The PRISMA 2020 statement: an updated guideline for reporting systematic reviews. BMJ. 2021;372:n71. 33782057 10.1136/bmj.n71 PMC8005924

[31] Peez C , Greßmann M , Raschke MJ , Glasbrenner J , Briese T , Frank A , et al. The bone bridge for tibial ACL graft fixation: a biomechanical analysis of different tibial fixation methods for ACL reconstruction. Orthop J Sports Med. 2023;11:23259671221143478. 36636032 10.1177/23259671221143478 PMC9830095

[32] Pontoh L , Dilogo I , Kamal A , Rhatomy S , Putra A , Fiolin J , et al. Anterior knee pain evaluation following anterior cruciate ligament (ACL) reconstruction using anterior half of the peroneus longus (AHPL) autograft. Orthop Res Rev. 2025;17:83–93. 39963304 10.2147/ORR.S495410 PMC11831479

[33] Samuelsen BT , Webster KE , Johnson NR , Hewett TE , Krych AJ . Hamstring autograft versus patellar tendon autograft for ACL reconstruction: is there a difference in graft failure rate? A meta‐analysis of 47,613 patients. Clin Orthop Relat Res. 2017;475:2459–2468. 28205075 10.1007/s11999-017-5278-9 PMC5599382

[34] Scheffler SU , Südkamp NP , Göckenjan A , Hoffmann RFG , Weiler A . Biomechanical comparison of hamstring and patellar tendon graft anterior cruciate ligament reconstruction techniques. Arthroscopy. 2002;18:304–315. 11877619 10.1053/jars.2002.30609

[35] Semnani‐Azad Z , Khan TA , Blanco Mejia S , de Souza RJ , Leiter LA , Kendall CWC , et al. Association of major food sources of fructose‐containing sugars with incident metabolic syndrome: a systematic review and meta‐analysis. JAMA Netw Open. 2020;3:e209993. 32644139 10.1001/jamanetworkopen.2020.9993 PMC7348689

[36] Shah A , Van Thiel G . Anterior cruciate ligament reconstruction with a biocomposite interference screw maintains graft fixation survival and improves clinical outcomes at 1 year: a multicenter prospective case series. Heliyon. 2023;9:e20921. 37867815 10.1016/j.heliyon.2023.e20921 PMC10585286

[37] Shea BJ , Reeves BC , Wells G , Thuku M , Hamel C , Moran J , et al. AMSTAR 2: a critical appraisal tool for systematic reviews that include randomised or non‐randomised studies of healthcare interventions, or both. BMJ. 2017;358:j4008. 28935701 10.1136/bmj.j4008 PMC5833365

[38] Slim K , Nini E , Forestier D , Kwiatkowski F , Panis Y , Chipponi J . Methodological index for non‐randomized studies (MINORS): development and validation of a new instrument. ANZ J Surg. 2003;73:712–716. 12956787 10.1046/j.1445-2197.2003.02748.x

[39] Sørensen OG , Faunø P , Konradsen L , Nielsen T , Schaarup S , Mygind‐Klavsen B , et al. Combined anterior cruciate ligament revision with reconstruction of the antero‐lateral ligament does not improve outcome at 2‐year follow‐up compared to isolated ACL revision; a randomized controlled trial. Knee Surg Sports Traumatol Arthrosc. 2023;31:5077–5086. 37733288 10.1007/s00167-023-07558-x PMC10598101

[40] Stang A . Critical evaluation of the Newcastle‐Ottawa scale for the assessment of the quality of nonrandomized studies in meta‐analyses. Eur J Epidemiol. 2010;25:603–605. 20652370 10.1007/s10654-010-9491-z

[41] Suomalainen P , Järvelä T , Paakkala A , Kannus P , Järvinen M . Double‐bundle versus single‐bundle anterior cruciate ligament reconstruction: a prospective randomized study with 5‐year results. Am J Sports Med. 2012;40:1511–1518. 22691456 10.1177/0363546512448177

[42] Teo WWT , Yeoh CSN , Wee THA . Tibial fixation in anterior cruciate ligament reconstruction. J Orthop Surg. 2017;25:2309499017699743. 10.1177/2309499017699743 28303744

[43] Tibor L , Chan PH , Funahashi TT , Wyatt R , Maletis GB , Inacio MCS . Surgical technique trends in primary ACL reconstruction from 2007 to 2014. J Bone Jt Surg. 2016;98:1079–1089. 10.2106/JBJS.15.00881 27385681

[44] Vieider RP , Berthold DP , Runer A , Winkler PW , Schulz P , Rupp MC , et al. The 50 most cited studies on posterior tibial slope in joint preserving knee surgery. J Exp Orthop. 2022;9:119. 36508044 10.1186/s40634-022-00557-w PMC9743935

[45] Weinstein JN , Tosteson TD , Lurie JD , Tosteson ANA , Hanscom B , Skinner JS , et al. Surgical vs nonoperative treatment for lumbar disk herniation: the Spine Patient Outcomes Research Trial (SPORT): a randomized trial. JAMA. 2006;296:2441–2450. 17119140 10.1001/jama.296.20.2441 PMC2553805

[46] Yari SS , El Naga AN , Patel A , Qadeer AA , Shah A . TightRope versus biocomposite interference screw for fixation in allograft ACL reconstruction: prospective evaluation of osseous integration and patient outcomes. JB JS Open Access. 2020;5:e0057. 33123662 10.2106/JBJS.OA.19.00057 PMC7418916

[47] Yıldırım C , Demirel M , Koraman E , Muratoğlu OG , Yamak F , Bozdağ SE , et al. The effect of supplementary staple fixation on biomechanical properties of soft tissue graft tibial fixation in anterior cruciate ligament reconstruction. J Knee Surg. 2024;37:736–741. 38599605 10.1055/s-0044-1786007

