# Supplementary material for: Impact of tibial supplementary fixation in anterior cruciate ligament reconstruction for soft tissue auto and allografts: Modest enhancement in stability and increased incidence of pain: A systematic review and meta‐analysis
Source: J Exp Orthop. 2025 Aug 5;12(3):e70390. doi: 10.1002/jeo2.70390 (PMC12322697; doi:10.1002/jeo2.70390)

Supporting Information

Title: Impact of tibial supplementary fixation in anterior cruciate ligament reconstruction for soft tissue auto and allografts: modest enhancement in stability and increased incidence of pain. A systematic review and meta-analysis

Journal of Experimental Orthopaedics

Fuwen Zheng, M.D.;1 Jiahao Gao, M.D.;1Chenyu Wang, M.D.;1 Xu Zheng, M.D.;2 Jinshuo Tang, M.D.; 1 Jinrui Zhang, M.D., Ph.D; 1, * Jianlin Zuo, M.D., Ph.D1, *

Jianlin Zuo, MD, PhD

Orthopaedic Surgeon, Professor in Orthopedics

Department of Orthopedics, China-Japan Union Hospital of Jilin University, Xiantai St No. 126, Changchun, Jilin Province, PR China. 130033, Tel: +8613514483288, E-mail: zuojl@jlu.edu.cn

Table S1. The detailed database of retrieval strategies

Table S2. Risk of Bias of Assessment for the Included RCTs Using the Cochrane Risk of Bias tool

Table S3. Newcastle-Ottawa Scale (NOS) for assessing the quality of cohort studies

Table S4. Methodological index for non-randomized studies (MINORS) for assessing the quality of cohort studies.

Table S5. Summary of complications between Group I and Group II in included RCTs

Table S6. GRADE Assessment

Figure S1. Forest plot of the subgroup results of SSD (<3 mm) on testing at 9.1 kg (20 lb) at 12 and 24 months

Figure S2. Forest plot of the subgroup results of SSD (<3 mm) on manual maximum testing at 24 months

Figure S3. Forest plot of the results of Pivot test at 8-12 months and 24 months

Figure S4. Forest plot of the results of Lachman test at 8-12 months

Figure S5. Forest plot of the results of IKDC objective grade at 24 months

Figure S6. Forest plot of the results of IKDC subjective score at 24 months

Figure S7. Forest plot of the subgroup results of re-tear between Group I and Group II after ACL reconstruction

Table S1. The detailed database of retrieval strategies

Pubmed

#1 “Anterior Cruciate Ligament”[Mesh]

#2 ((((((((((((Anterior Cruciate Ligament[Title/Abstract]) OR (Ligament, Anterior Cruciate[Title/Abstract])) OR (Cruciate Ligaments, Anterior[Title/Abstract])) OR (Ligaments, Anterior Cruciate[Title/Abstract])) OR (Anterior Cruciate Ligaments[Title/Abstract])) OR (Cruciate Ligament, Anterior[Title/Abstract])) OR (Anterior Cranial Cruciate Ligament[Title/Abstract])) OR (Ligament, Cranial Cruciate[Title/Abstract])) OR (Cranial Cruciate Ligament[Title/Abstract])) OR (Cranial Cruciate Ligaments[Title/Abstract])) OR (Ligaments, Cranial Cruciate[Title/Abstract])) OR (Cruciate Ligaments, Cranial[Title/Abstract])) OR (Cruciate Ligament, Cranial[Title/Abstract])

#3 #1 OR #2

#4 (supplement* [Title/Abstract]) OR (second* [Title/Abstract])

#5 fixation*[Title/Abstract]

#6 #3 AND #4 AND #5

Embase

#1’anterior cruciate ligament’/exp or ‘anterior cruciate knee ligament’:ti,ab or ‘anterior cruciate ligament, knee’:ti,ab or ‘cruciate knee ligament, anterior’:ti,ab or ‘ligament, anterior cruciate knee’:ti,ab or ‘anterior cruciate ligament’:ti,ab

#2 ‘supplement*’:ti,ab or ‘second*’:ti,ab

#3 ‘fixation*’:ti,ab

#4 #1 AND #2 AND #3

Cochrane Library

#1 MeSH descriptor: [Anterior Cruciate Ligament] explode all trees

#2 (Anterior Cruciate Ligament):ti,ab,kw OR (Ligament, Anterior Cruciate):ti,ab,kw OR (Cruciate Ligaments, Anterior):ti,ab,kw OR (Ligaments, Anterior Cruciate):ti,ab,kw OR (Anterior Cruciate Ligaments):ti,ab,kw OR (Cruciate Ligament, Anterior):ti,ab,kw OR (Anterior Cranial Cruciate Ligament):ti,ab,kw OR (Ligament, Cranial Cruciate):ti,ab,kw OR (Cranial Cruciate Ligament):ti,ab,kw OR (Cranial Cruciate Ligaments) :ti,ab,kw OR (Ligaments, Cranial Cruciate) :ti,ab,kw OR (Cruciate Ligaments, Cranial) :ti,ab,kw OR (Cruciate Ligament, Cranial) :ti,ab,kw

#3 #1 OR #2

#4 (supplement*):ti,ab,kw OR (second*):ti,ab,kw

#5 (fixation*):ti,ab,kw

#6 #3 AND #4 AND #5

Web of Science

1: (((((((((((TS=(Anterior Cruciate Ligament)) OR TS=(Cruciate Ligament, Anterior)) OR TS=(Anterior Cruciate Ligaments)) OR

TS=(Cruciate Ligaments, Anterior)) OR TS=(Ligament, Anterior Cruciate)) OR TS=(Ligaments, Anterior Cruciate)) OR TS=(Anterior Cranial Cruciate Ligament)) OR TS=(Cranial Cruciate Ligament)) OR TS=(Cranial Cruciate Ligaments)) OR TS=(Cruciate Ligament, Cranial)) OR TS=(Cruciate Ligaments, Cranial)) OR TS=(Ligament, Cranial Cruciate)) OR TS=(Ligaments, Cranial Cruciate)

2: (TS=(supplement*)) OR TS=(second*)

3: TS=(fixation*)

4: #1 AND #2 AND #3

Table S2. Risk of Bias of Assessment for the Included RCTs Using the Cochrane Risk of Bias tool

Author, year	Random sequence generation	Allocation concealment	Blinding of participants and personnel	Blinding of outcome assessment	Incomplete outcome data	Selective reporting	Other bias
Mousavi et al, 2020	+	+	+	+	+	+	+
Carulli et al, 2017	+	+	+	+	+	+	+
Noh et al, 2012	+	?	+	+	+	+	+
De Wall et al, 2011	?	+	+	+	+	+	+
Hill et al, 2005	+	+	+	+	+	+	+
**Note:** + = Low risk of bias, **?** = Unclear risk of bias, **-** = High risk of bias.							

Table S3. Newcastle-Ottawa Scale (NOS) for assessing the quality of cohort studies

Author, year	Selection[a]				Comparability[b]		Outcome[c]			Score[d]
Abudaqqa et al, 2023	*	*	*	*	*	-	-	*	*	7
Teo et al, 2017	*	*	*	*	*	*	-	-	*	7
Lim et al, 2009	*	*	*	*	*	-	*	*	*	8

Note:

a, 4 points at most may be given for the representativeness of the cohort, the choice of a non-exposed cohort, assessment of exposure, and confirmation that the baseline outcome was absent

b, 2 points at most are available for controlling the pre-specified confounding variable (surgeon) and other confounding variables

c, 3 points at most can be awarded for outcome assessment, follow-up duration (≥ 2 years), and the adequacy of follow-up

d, 9 points at most could be awarded

Table S4. Methodological index for non-randomized studies (MINORS) for assessing the quality of cohort studies.

Items	Abudaqqa et al,2023	Teo et al,2017	Lim et al 2009
A clearly stated aim	2	2	2
Inclusion of consecutive patients	2	2	2
Prospective collection of data	0	0	0
Endpoints appropriate to the aim of the study	2	2	2
Unbiased assessment of the study endpoint	1	1	2
Follow-up period appropriate to the aim of the study	1	0	2
Loss to follow up less than 5%	2	2	2
Prospective calculation of the study size	2	2	2
An adequate control group	2	2	2
Contemporary groups	2	2	2
Baseline equivalence of groups	1	1	2
Adequate statistical analyses	0	0	0
Total scores	17	16	20

Note：

1.The items are scored 0 (not reported), 1 (reported but inadequate) or 2 (reported and adequate). The global ideal score being 16 for non-comparative studies and 24 for comparative studies.

2.A score greater than or equal to 16 points in comparative studies or a score greater than or equal to 11 points in non-comparative studies is considered high-quality research.

Table S5 Summary of complications between Group I and Group II in included RCTs

**Author, year**	**Complications**	
	**Group I**	**Group II**
Hill et al, 2005	6 kneeling pain	2 kneeling pain
Lim et al, 2009	9 thigh atrophy	13 thigh atrophy; one 10° loss of flexion
De Wall et al, 2011	2 retear	5 retear
Noh et al, 2012	0 skin irritation, tendernes, or other complications	0 skin irritation, tenderness, or other complications
Teo et al, 2017	4 kneeling pain	0 kneeling pain
Carulli et al, 2017	3 wound infections; 2 effusion and persistent pain; 1 retear; 2 late infections; 5 kneeling pain	1 rupture of the sheath; 2 wound infections; 2 effusion and persistent pain; 1 retear
Mousavi et al, 2020	0 complication	0 complication
Abudaqqa et al 2023	45 complications; 4 retear	80 complications; 13 retear

Note:

Group I: screw interference with supplementary fixation; **Group II**: screw interference alone;

Table S6. GRADE Assessment

Certainty assessment							№ of patients		Effect		Certainty
**№ of studies**	**Study design**	**Risk of bias**	**Inconsistency**	**Indirectness**	**Imprecision**	**Other considerations**	**Group I**	**Group II**	**Relative (95% CI)**	**Absolute (95% CI)**	
**SSD: screw vs. screw/staples – Follow-up 24 months**											
2	RCTs	not serious	serious[a]	not serious	not serious[e]	none	56	63	-	MD **1.02 lower** (1.79 lower to 0.25 lower)	⊕⊕⊕◯ Moderate
**SSD: screw/sheath vs. screw/staples – Follow-up 24 months**											
2	RCTs	not serious	not serious	not serious	serious[f]	none	91	89	-	MD **0.09 higher** (0.03 lower to 0.22 higher)	⊕⊕⊕◯ Moderate
**Subgroup of SSD (<3 mm) on testing at 9.1 kg (20 lb) – Follow-up 12 months**											
1	non-RCTs	not serious	undetected[b]	serious[d]	serious[g]	none	31/33 (93.9%)	29/31 (93.5%)	**RR 1.00** (0.88 to 1.14)	**0 fewer per 1,000** (from 112 fewer to 131 more)	⊕◯◯◯ Very low
**Subgroup of SSD (<3 mm) on testing at 9.1 kg (20 lb) – Follow-up 24 months**											

2	RCTs	not serious	not serious	not serious	serious[g]	none	33/36 (91.7%)	34/43 (79.1%)	**RR 1.17** (0.97 to 1.41)	**134 more per 1,000** (from 24 fewer to 324 more)	⊕⊕⊕◯ Moderate
**Subgroup of SSD (<3 mm) on manual maximum testing – Follow-up 24 months**											
2	RCTs	not serious	not serious	not serious	serious[g]	none	30/36 (83.3%)	28/43 (65.1%)	**RR 1.29** (0.99 to 1.68)	**189 more per 1,000** (from 7 fewer to 443 more)	⊕⊕⊕◯ Moderate
**Pivot test – Follow-up 8-12 months**											
2	non-RCTs	not serious	not serious	not serious	serious[g]	none	6/55 (10.9%)	5/61 (8.2%)	**RR 1.35** (0.44 to 4.15)	**29 more per 1,000** (from 46 fewer to 258 more)	⊕◯◯◯ Very low
**Pivot test – Follow-up 24 months**											
2	RCTs	not serious	not serious	not serious	serious[g]	strong association[j]	6/56 (10.7%)	15/63 (23.8%)	**RR 0.43** (0.18 to 1.01)	**136 fewer per 1,000** (from 195 fewer to 2 more)	⊕⊕⊕⊕ High

**Lachman test – Follow-up 8-12 months**											
2	non-RCTs	not serious	not serious	not serious	serious[g]	None	3/55 (5.5%)	4/61 (6.6%)	**RR 0.84** (0.22 to 3.15)	**10 fewer per 1,000** (from 51 fewer to 141 more)	⨁◯◯◯ Very low
**Lachman test – Follow-up 24 months**											
2	RCTs	not serious	not serious	not serious	not serious[h]	strong association[j]	10/56 (17.9%)	26/63 (41.3%)	**RR 0.43** (0.23 to 0.81)	**235 fewer per 1,000** (from 318 fewer to 78 fewer)	⨁⨁⨁⨁ High
**IKDC objective grade – Follow-up 24 months**											
2	RCTs	not serious	not serious	not serious	serious[g]	none	5/56 (8.9%)	11/63 (17.5%)	**RR 0.52** (0.19 to 1.39)	**84 fewer per 1,000** (from 141 fewer to 68 more)	⨁⨁⨁◯ Moderate
**IKDC subjective score – Follow-up 24 months**											

2	RCTs	not serious	serious[b]	not serious	serious[f]	none	91	89	-	MD **1.42 higher** (2.08 lower to 4.92 higher)	⊕⊕◯◯ Low
**Major complications – kneeling pain**											
4	non-RCTs	not serious	not serious	not serious	not serious[i]	very strong association[k]	15/134 (11.2%)	2/139 (1.4%)	**RR 6.01** (1.79 to 20.22)	**72 more per 1,000** (from 11 more to 177 more)	⊕⊕⊕⊕ High
**Major complications: screw vs. screw/staples – ACL retear**											
2	non-RCTs	not serious	not serious	not serious	serious[g]	none	4/191 (2.1%)	13/358 (3.6%)	**RR 0.61** (0.20 to 1.84)	**14 fewer per 1,000** (from 29 fewer to 31 more)	⊕◯◯◯ Very low
**Major complications: screw/sheath vs. screw/staples – ACL retear**											
2	RCTs	not serious	not serious	not serious	serious[g]	strong association[j]	3/99 (3.0%)	6/98 (6.1%)	**RR 0.49** (0.13 to 1.91)	**31 fewer per 1,000** (from 53 fewer to 56 more)	⊕⊕⊕⊕ High

Group I: screw interference with supplementary fixation; **Group II**: screw interference alone; **CI**: confidence interval; **RCTs**: randomized controlled trials; **SSD**: side-to-side difference; **MD**: mean difference; **RR**: risk ratio; **IKDC**: International Knee Documentation Committee

Explanations

a. Despite observing substantial inter-study heterogeneity ($I^2 = 51\%$), the estimates were uniformly aligned and showed considerable overlap, leading us to conclude that this was not a serious inconsistency.

b. Inconsistency assessment was not possible as only one study was included.

c. Downgrade for serious inconsistency, as the high heterogeneity between the two studies ($I^2 = 84\%$).

d. Downgrade for serious indirectness was warranted due to only one cohort being available, which limits the generalizability to the general population.

e. No downgrade for serious imprecision was applied, as the upper bound of the 95% CI for the MD is below 0.

f. Downgrade for serious imprecision, as the 95% CI for the MD includes values both below and above 0.

g. Downgrade for serious imprecision, as the 95% CI for the RR includes the threshold for clinically unimportant effects (RR 0.95 – 1.05).

h. No downgrade for serious imprecision was applied, as the upper bound of the 95% CI for the RR does not include the range for clinically unimportant effects (RR 0.95 – 1.05).

i. No downgrade for serious imprecision was applied, as the lower bound of the 95% CI for the RR does not include the range for clinically unimportant effects (RR 0.95 – 1.05).

j. Upgrade the quality of evidence by 1 level for RR either >2.0 or <0.5 based on consistent evidence from at least 2 studies.

k. Upgrade the quality of evidence by 2 level for RR either >5.0 or <0.2 based on consistent evidence from at least 2 studies.

Figure S1. Forest plot of the subgroup of SSD (<3 mm) on testing at 9.1 kg (20 lb) at 12 and 24 months

	Group I		Group II			Odds Ratio
Study or Subgroup	Events	Total	Events	Total	Weight	M-H, Fixed, 95% CI
**1.1.1 Follow-up 12 months**						
Teo 2017	31	33	29	31	100.0%	1.07 [0.14, 8.09]
**Subtotal (95% CI)**		**33**		**31**	**100.0%**	**1.07 [0.14, 8.09]**
Total events	31		29			
Heterogeneity: Not applicable						
Test for overall effect: Z = 0.06 (P = 0.95)						
**1.1.2 Follow-up 24 months**						
Lim 2009	14	15	10	16	24.4%	8.40 [0.87, 81.08]
Hill 2005	19	21	24	27	75.6%	1.19 [0.18, 7.84]
**Subtotal (95% CI)**		**36**		**43**	**100.0%**	**2.95 [0.75, 11.51]**
Total events	33		34			
Heterogeneity: $Chi^2 = 1.71$, df = 1 (P = 0.19); $I^2 = 42\%$						
Test for overall effect: Z = 1.55 (P = 0.12)						

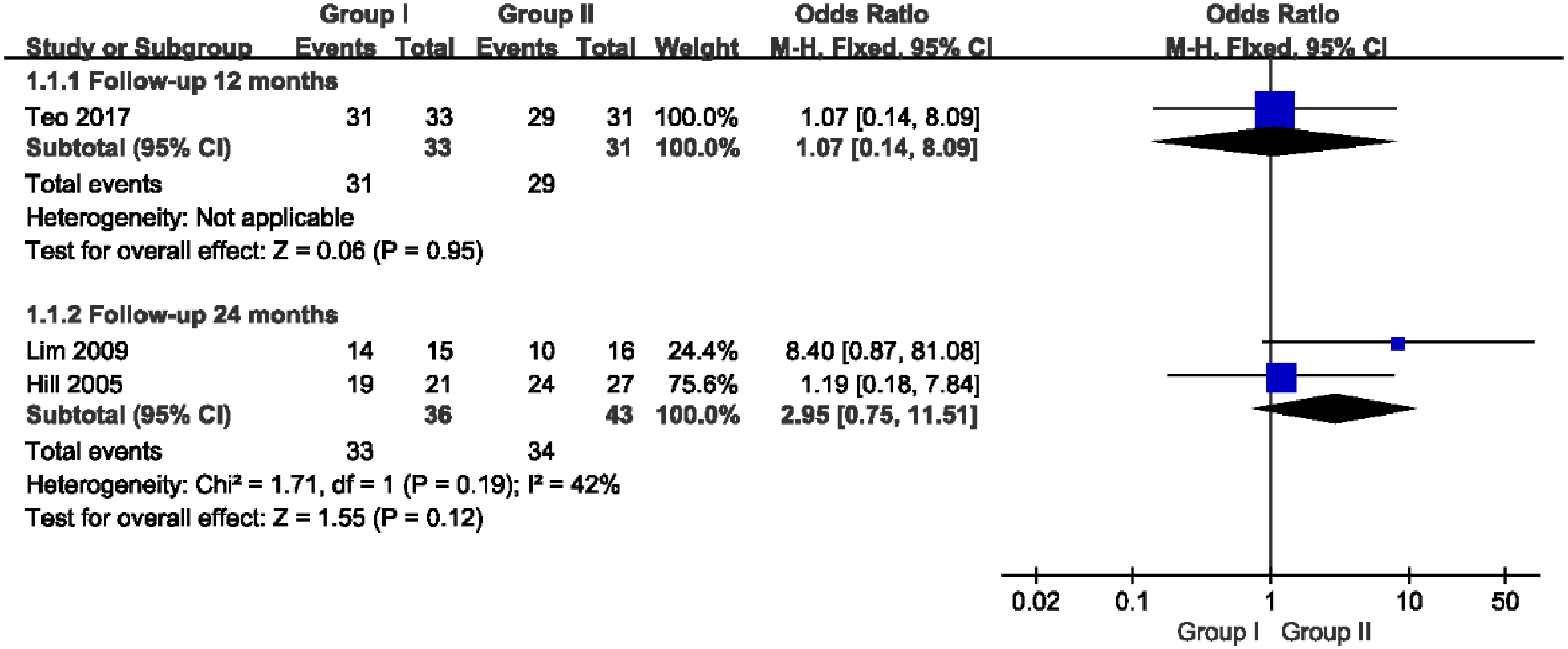

Figure S2. Forest plot of the subgroup of SSD (<3 mm) on manual maximum testing at 24 months

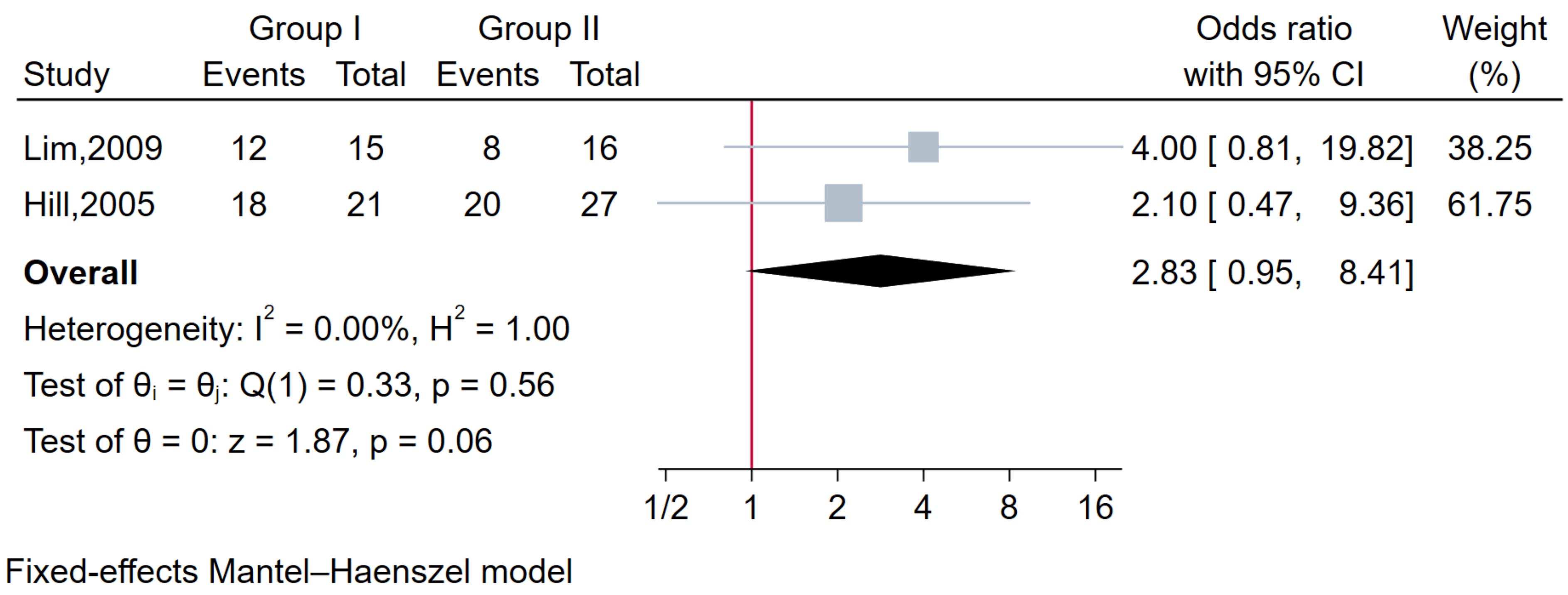

Figure S3. Forest plot of Pivot test at 8-12 months and 24 months

	Group I		Group II			Odds Ratio	
Study or Subgroup	Events	Total	Events	Total	Weight	M-H, Fixed, 95% CI	Year
**3.1.1 Follow-up 8-12 months**							
Mousavi 2020	4	24	3	28	56.0%	1.67 [0.33, 8.32]	2020
Teo 2017	2	31	2	33	44.0%	1.07 [0.14, 8.09]	2017
**Subtotal (95% CI)**		**55**		**61**	**100.0%**	**1.40 [0.40, 4.92]**	
Total events	6		5				
Heterogeneity: Chi² = 0.11, df = 1 (P = 0.74); I² = 0%							
Test for overall effect: Z = 0.53 (P = 0.60)							
**3.1.2 Follow-up 24 months**							
Noh 2012	5	35	12	36	80.2%	0.33 [0.10, 1.08]	2012
Hill 2005	1	21	3	27	19.8%	0.40 [0.04, 4.15]	2005
**Subtotal (95% CI)**		**56**		**63**	**100.0%**	**0.35 [0.12, 0.99]**	
Total events	6		15				
Heterogeneity: Chi² = 0.02, df = 1 (P = 0.89); I² = 0%							
Test for overall effect: Z = 1.98 (P = 0.05)							

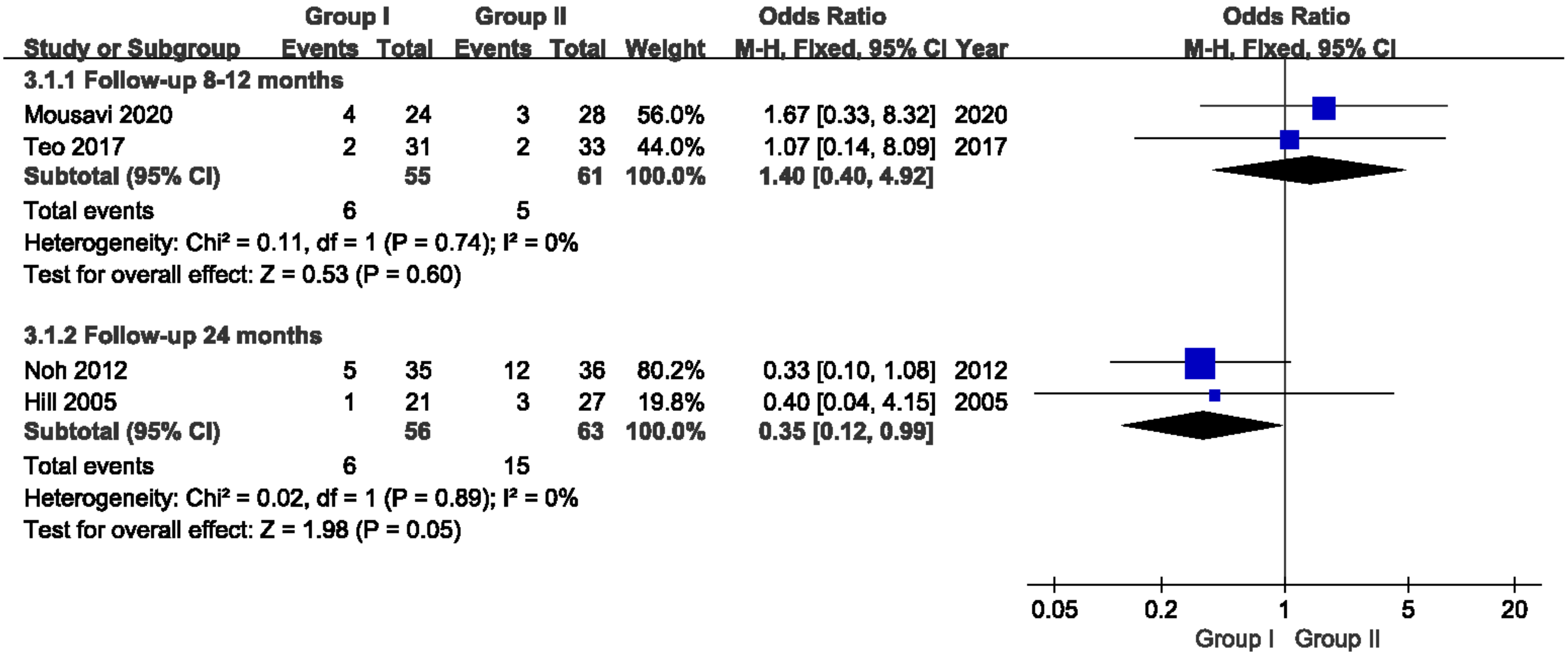

Figure S4. Forest plot of Lachman test at 8-12 months

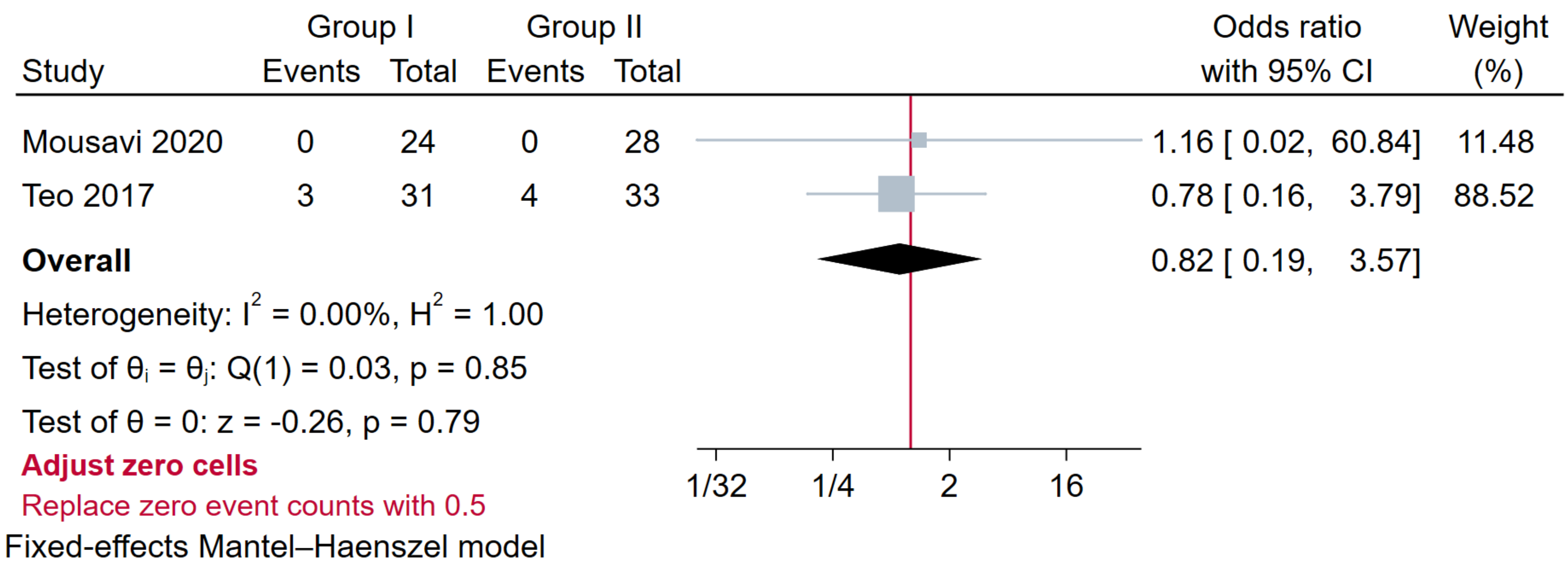

Figure S5. Forest plot of IKDC objective grade at 24 months

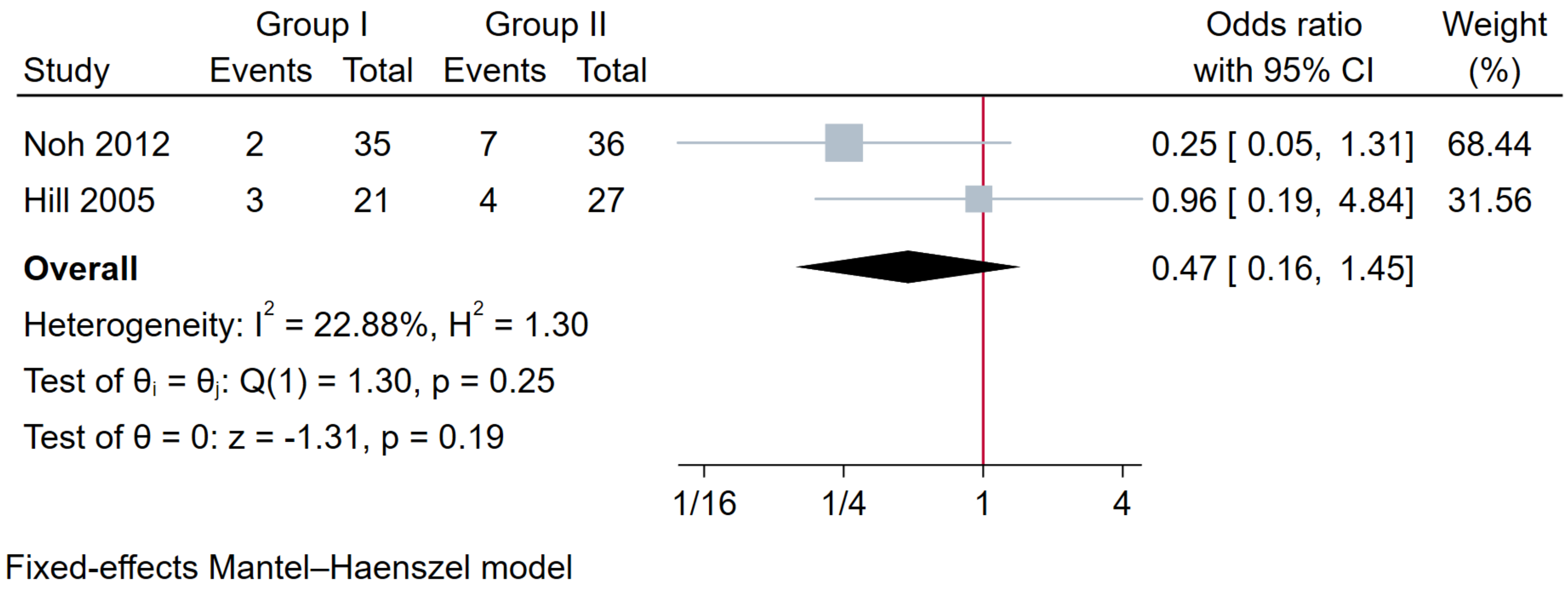

Figure S6. Forest plot of IKDC subjective score at 24 months

	Group I			Group II				Mean Difference
Study or Subgroup	Mean	SD	Total	Mean	SD	Total	Weight	IV, Random, 95% CI
Carulli 2017	92	1.9	45	89	2.7	45	56.1%	3.00 [2.04, 3.96]
De Wall 2011	76.1	6.9	46	76.7	5.9	44	43.9%	-0.60 [-3.25, 2.05]
**Total (95% CI)**			**91**			**89**	**100.0%**	**1.42 [-2.08, 4.92]**

Heterogeneity: Tau² = 5.45; Chi² = 6.27, df = 1 (P = 0.01); I² = 84%
Test for overall effect: Z = 0.79 (P = 0.43)

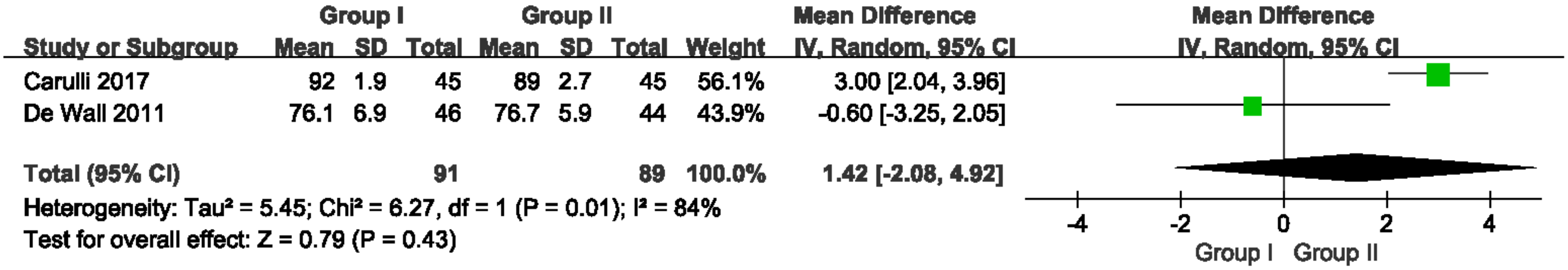

Figure S7. Forest plot of the subgroup results of re-tear between Group I and Group II after ACL reconstruction

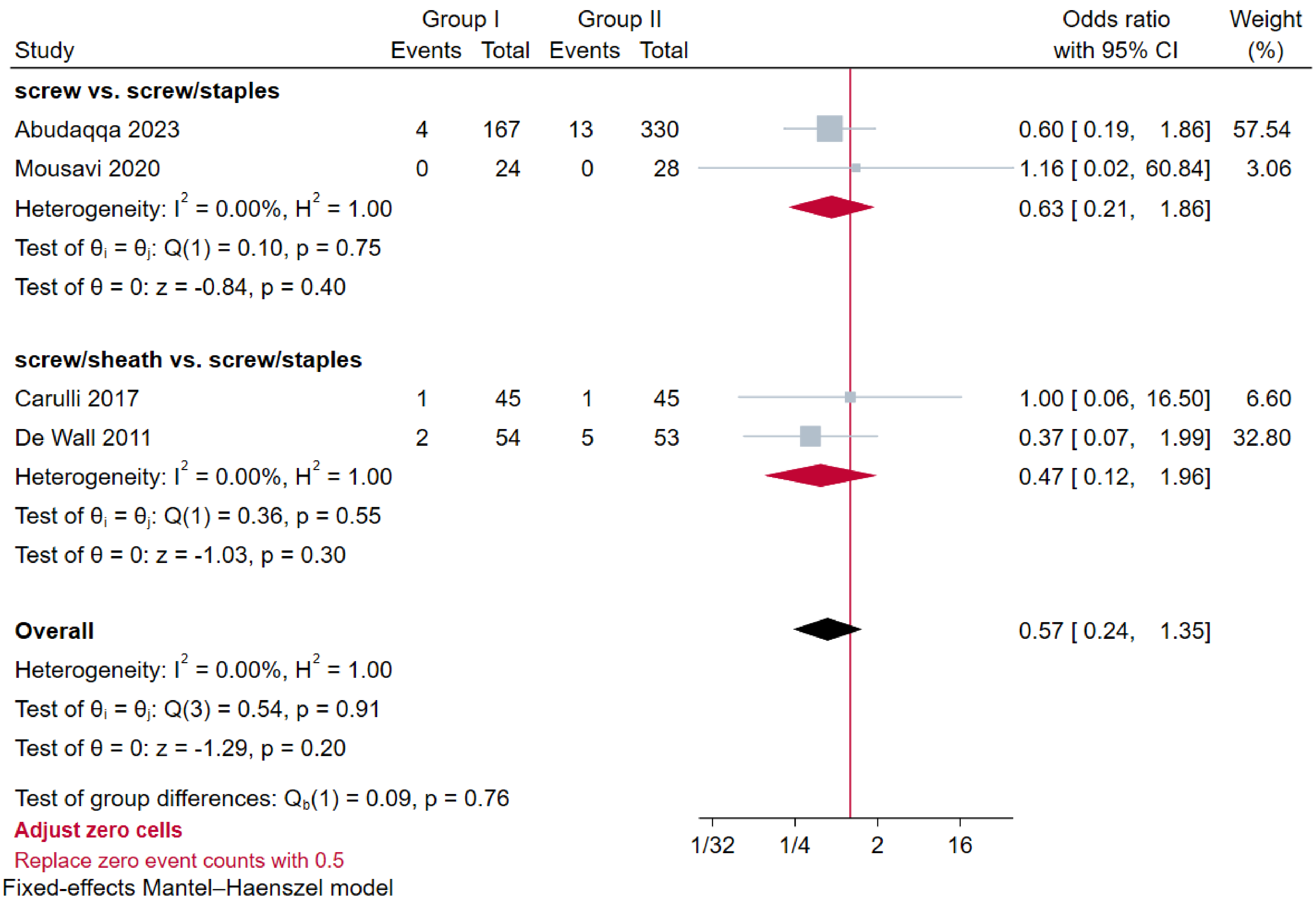

Supplement: Supplementary file 1 — Supporting information. [file JEO2-12-e70390-s001.pdf]
